# Mean arterial pressure ≥89 mmHg before 20+0 weeks of gestation as a predictor of early-onset preeclampsia: a case-control study

**DOI:** 10.11604/pamj.2026.54.26.52221

**Published:** 2026-06-03

**Authors:** Willis Okumu Ochieng, Margaret Kamene Kilonzo, Rosa Ndiema Chemwey, Omondi Ogutu

**Affiliations:** 1 Department of Obstetrics and Gynaecology, University of Nairobi, Nairobi, Kenya,; 2 Department of Obstetrics and Gynaecology, Kenyatta National Hospital, Nairobi, Kenya

**Keywords:** Early-onset preeclampsia, mean arterial pressure, predictive performance, screening, low-and middle-income countries

## Abstract

**Introduction:**

early-onset preeclampsia (EOPE) contributes substantially to maternal and perinatal morbidity in low- and middle-income countries (LMICs). Mean arterial pressure (MAP) has been evaluated as a potential predictor of EOPE; however, its predictive performance before 20+0 weeks of gestation remains unclear in LMIC settings. This study evaluated the predictive performance of MAP ≥89 mmHg measured before 20+0 weeks in predicting EOPE among at-risk women at Kenyatta National Hospital (KNH)

**Methods:**

an unmatched case-control study was conducted at KNH, including 582 women (194 cases, 388 controls) with at least one preeclampsia risk factor identified before 20+0 weeks. Recorded blood pressure measurements were used to calculate MAP. Predictive performance was assessed using sensitivity, specificity, and receiver operating characteristic (ROC) curve analysis, with the area under the ROC curve (AUROC) reported. Multivariable logistic regression was used to assess independent associations.

**Results:**

median MAP was higher among EOPE cases than controls (94.56 mmHg (IQR: 87.00-104.58) vs 83.33 mmHg (IQR: 77.67-89.83); p <0.001). Mean arterial pressure ≥89 mmHg was associated with EOPE (OR = 5.66, 95% CI: 3.89-8.24, p <0.001), and remained an independent predictor (aOR = 3.04, 95% CI: 1.96-4.71, p <0.001). Discriminatory performance was fair (AUROC = 0.783) and improved when combined with maternal risk factors (AUROC = 0.810). Sensitivity and specificity were 68.04% and 72.68%, respectively, while the positive and negative predictive values were 55.46% and 81.98%, respectively.

**Conclusion:**

mean arterial pressure ≥89 mmHg before 20+0 weeks independently predicts EOPE among at-risk women and may support early risk stratification in LMIC settings.

## Introduction

Hypertensive disorders of pregnancy (HDPs) affect 5-10% of pregnancies and remain an important cause of maternal and perinatal morbidity and mortality globally. The burden is disproportionately higher in sub-Saharan Africa, where severe disease at presentation is common and most preeclampsia-related maternal deaths occur in low- and middle-income countries (LMICs) [1,2]. Preeclampsia occurring before 34+0 weeks of gestation is classified as early-onset preeclampsia (EOPE), while late-onset preeclampsia (LOPE) develops at or beyond 34+0 weeks. Early-onset preeclampsia is associated with more severe disease and adverse perinatal complications compared with LOPE. Additionally, established risk factors for preeclampsia are more frequently observed in women with EOPE [2,3]. Early identification of women at risk during antenatal care can significantly reduce poor outcomes. However, this remains a challenge in LMIC settings due to inadequately implemented preventive measures. Despite the high burden of disease, high-quality evidence from these settings remains limited. Several methods for predicting preeclampsia have been explored; however, most have limited reliability. Mean arterial pressure (MAP), derived from standard blood pressure measurements, has been investigated as a potential predictive marker. Some studies have shown that elevated MAP in the mid-trimester is associated with increased risk of preeclampsia, although its discriminatory performance is modest [4,5]. However, data on MAP assessed before 20+0 weeks remain scarce. This study therefore aimed to evaluate the sensitivity, specificity, and predictive values of MAP measured before 20+0 weeks of gestation for early-onset preeclampsia (EOPE), stratified by maternal risk factors, and to assess its independent predictive ability after adjusting for confounders.

## Methods

**Study design and setting:** this unmatched case-control study was conducted among women seeking antenatal, maternity, and postnatal services at Kenyatta National Hospital (KNH), a national teaching and referral hospital in Nairobi with a capacity of 1,800 beds. The hospital serves patients from Nairobi and surrounding counties.

**Study period:** this study was conducted between March 2025 and July 2025.

**Inclusion criteria:** women aged 18-49 years with at least one documented risk factor for preeclampsia, at least one recorded blood pressure measurement prior to 20+0 weeks of gestation, and initiation of antenatal care before 20+0 weeks of gestation were eligible. Cases were defined as women diagnosed with preeclampsia before 34+0 weeks of gestation. Preeclampsia was defined as new-onset hypertension (≥140/90 mmHg) after 20+0 weeks of gestation accompanied by proteinuria (≥300 mg in a 24-hour urine collection, or protein/creatinine ratio ≥0.3, or dipstick ≥1+) or evidence of maternal end-organ dysfunction [6,7]. Controls were women who did not meet the criteria for preeclampsia. Among women with chronic hypertension, cases were those who developed new-onset thrombocytopenia, renal or hepatic dysfunction, or clinical features suggestive of preeclampsia. Women with chronic hypertension who did not develop these features were classified as controls [6,7]. Controls were selected from the same source population as cases, namely women attending antenatal, maternity, and postnatal services at Kenyatta National Hospital during the study period, to ensure comparability. The use of consecutive recruitment for both cases and controls minimized selection bias and ensured that controls were representative of the population at risk from which cases arose. Eligible women were consecutively recruited and interviewed by trained obstetrics and gynaecology residents serving as research assistants. Uniformity was ensured by training all research assistants on the study protocol and standardized data collection procedures.

**Exclusion criteria:** women who declined or could not provide informed consent were excluded. Participants with incomplete antenatal records before 20+0 weeks of gestation, or those with missing blood pressure measurements required for mean arterial pressure (MAP) calculation, were also excluded to ensure accurate exposure assessment.

**Study variables:** all study variables were obtained through structured participant interviews and retrospective review of antenatal care (ANC) records and inpatient files. In addition to preeclampsia and mean arterial pressure (MAP), the following variables were collected: maternal age ≥35 years (yes/no), low income (yes/no), nulliparity (yes/no), multifetal gestation (yes/no), interpregnancy interval >10 years (yes/no), family history of preeclampsia (sister/mother) (yes/no), previous adverse pregnancy outcome or small-for-gestational-age (SGA) infant (yes/no), assisted reproductive technology (ART) (yes/no), previous preeclampsia (yes/no), pregestational diabetes mellitus (PGDM) (yes/no), chronic hypertension (yes/no), renal disease (yes/no), autoimmune disease (yes/no), aspirin prophylaxis (yes/no), and calcium prophylaxis (yes/no). Low income was defined as a monthly household income of ≤KES 46,355 [8]. Structured interviews were used to obtain sociodemographic data and selected clinical history, while clinical data, including systolic and diastolic blood pressure (SBP and DBP), obstetric history, and medical conditions prior to 20+0 weeks of gestation, were extracted from ANC records and inpatient files.

**Exposure assessment:** mean arterial pressure ≥89 mmHg was the main exposure variable. Mean arterial pressure was calculated using the formula: MAP = DBP + (SBP - DBP)/3 [5]. Only blood pressure measurements recorded before 20+0 weeks of gestation were included in the analysis. The threshold of MAP ≥89 mmHg was selected a priori based on previously reported cut-offs in the literature [5], rather than deriving an optimal cut-off from the study data. This approach minimized overfitting and optimistic bias associated with data-driven thresholds and enhanced comparability with existing studies and external validity.

**Outcome measure:** early-onset preeclampsia (EOPE), the primary outcome, was defined as preeclampsia diagnosed before 34+0 weeks of gestation [2,3], based on ANC records and inpatient files. Gestational age was confirmed using last menstrual period (LMP) or first- or second-trimester ultrasonography [9].

**Sample size and statistical power:** the required sample size was calculated using Kelsey *et al*. formula for unmatched case-control studies [10], assuming 80% power, a 95% confidence interval (CI), and an expected odds ratio (OR) of 1.7 derived from previous studies [11], and a 1: 2 case-to-control ratio. Based on these assumptions, a minimum sample size of 194 cases and 388 controls was obtained. This sample size provided 80% power to detect a 1.7-fold increase in the odds of EOPE associated MAP.

**Statistical methods:** data were entered and cleaned in Microsoft Excel LTSC Professional Plus 2021, then coded and analyzed using IBM SPSS Version 27.0.1. Continuous variables, including MAP, maternal age, and gestational age at first antenatal contact, were summarized using medians and interquartile ranges (IQRs) due to non-normal distribution. Normality was assessed using the Shapiro-Wilk test and visual inspection of histograms. Comparisons between EOPE and non-EOPE groups were performed using the Mann-Whitney U test. Categorical variables were summarized as frequencies and percentages, and compared using the Chi-square or Fisher´s exact tests, as appropriate. Receiver operating characteristic (ROC) curve analysis using continuous MAP values was performed to assess discriminatory ability, and the area under the ROC curve (AUROC) was reported [12]. Sensitivity, specificity, positive predictive value (PPV), and negative predictive value (NPV) for the MAP cut-off of ≥89 mmHg were subsequently calculated. Subgroup analyses were performed by stratifying according to predefined maternal risk factors as listed under study variables, with corresponding measures of predictive performance calculated. Bivariate analysis was conducted to examine associations between each risk factor and EOPE, with crude odds ratios (ORs) and 95% confidence intervals (CIs) reported. Multivariable logistic regression was used to adjust for potential confounders. All variables of clinical relevance, including predefined maternal risk factors and the primary exposure (MAP ≥89 mmHg), were included in the model irrespective of their significance in bivariate analysis. Adjusted odds ratios (aORs) with 95% confidence intervals (CIs) were reported, and model discrimination was assessed using AUROC [12]. A p-value <0.05 was considered statistically significant. All statistical tests were two-sided. For multivariable analysis, variables related to previous pregnancies (previous adverse pregnancy outcomes or small-for-gestational-age infant, interpregnancy interval, and previous preeclampsia) were recoded as “No” for primigravid women, as these variables were not applicable to this subgroup. No imputation was performed, as there were no missing data for the variables included in the analysis.

**Bias:** potential selection bias may have arisen from the hospital-based case-control design and inclusion of women with pre-existing risk factors for preeclampsia. Information bias may have resulted from retrospective record review and variability in blood pressure measurement techniques, while recall bias may have occurred for interview-derived data. Misclassification bias was minimized through standardized EOPE definitions and a structured data abstraction tool. Confounding was addressed using multivariable logistic regression.

**Ethical consideration:** this study was approved by Kenyatta National Hospital-University of Nairobi Ethics Review Committee (KNH-UoN ERC) (protocol number: P781/10/2024), National Commission for Science, Technology and Innovation (NACOSTI) (license number: 112107), and the KNH Department of Obstetrics and Gynaecology (reference: KNH/HOD-OBS&GYN/51/VOL.111). All participants were informed about the study purpose, procedures, and duration. Written informed consent was obtained prior to participation, and participants´ questions were addressed before enrolment.

## Results

**Participant characteristics and mean arterial pressure distribution before 20+0 weeks of gestation:** a total of 883 participants (285 cases and 598 controls) from the antenatal, maternity, and postnatal units at Kenyatta National Hospital (KNH) were consecutively assessed for eligibility during the study period. Of these, 301 were excluded: 279 (83 cases and 196 controls) did not meet the inclusion criteria, and 22 (8 cases and 14 controls) declined consent. Consequently, 582 participants (194 cases and 388 controls) were included in the final analysis. Participants were approached while awaiting services in these units. Those who consented were interviewed, and their mean arterial pressure (MAP) measurements before 20+0 weeks of gestation were extracted from antenatal care records. Figure 1 describes the participant flow through the study. The median MAP before 20+0 weeks was 86.67 mmHg (IQR: 80.17-94.00). Mean arterial pressure was significantly higher among EOPE cases compared with controls (94.56 mmHg (IQR: 87.00-104.58) vs 83.33 mmHg (IQR: 77.67-89.83); p <0.001). The median gestational age at first documented blood pressure measurement was 14 weeks (IQR: 10-18), slightly higher among cases (16 weeks; IQR: 12-18) than controls (14 weeks; IQR: 10-17). Median maternal age was 28 years (IQR: 23-35), higher among cases (30 years; IQR: 26-35) than controls (27 years; IQR: 22-35). EOPE cases were more likely than controls to have MAP ≥89 mmHg, previous preeclampsia, chronic hypertension, aspirin prophylaxis, kidney disease, and family history of preeclampsia (sister/mother). Similarly, MAP ≥89 mmHg was more frequent among women with previous preeclampsia, chronic hypertension, aspirin prophylaxis, family history of preeclampsia (sister/mother), kidney disease, maternal age ≥35 years, and pregestational diabetes mellitus (PGDM). Nulliparity was less common among EOPE cases and among women with MAP ≥89 mmHg. Table 1 summarizes baseline participant characteristics.

**Figure 1:**
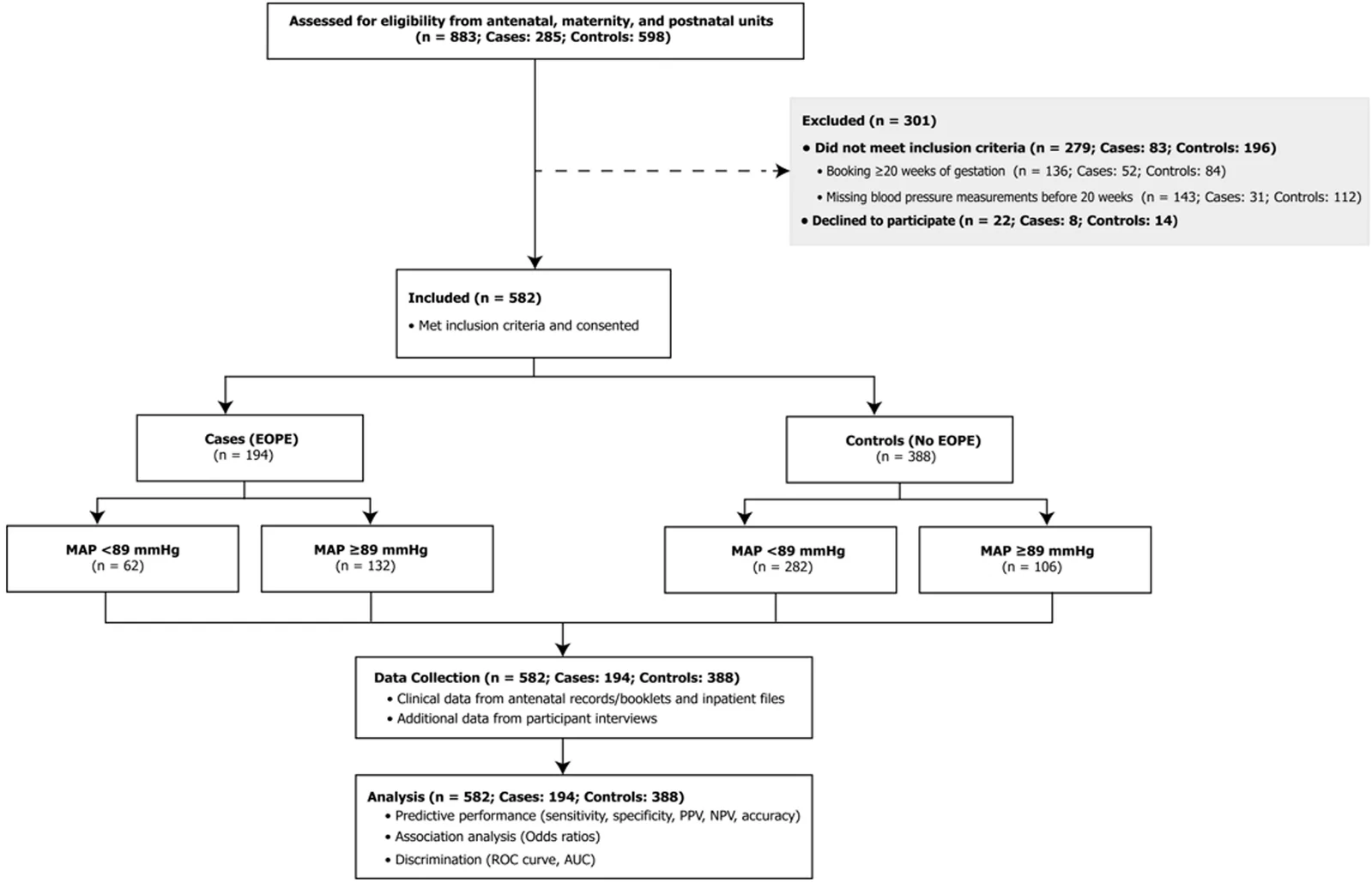
flow diagram for study participants

**Table 1:** baseline participant characteristics and maternal risk factors by early-onset preeclampsia status and mean arterial pressure ≥89 mmHg

Variable	No EOPE (%) (N = 388)	EOPE (%) (N = 194)	MAP <89 mmHg (%) (N = 344)	MAP ≥89 mmHg (%) (N = 238)	Total (%) (N = 582)	P-value (EOPE)	P-value (MAP)
**Continous variables**							
Average MAP <20+0 weeks, median (IQR)	83.33 mmHg (IQR: 77.67-89.83)	94.56 mmHg (IQR: 87.00-104.58)	-	-	86.67 mmHg (IQR: 80.17-94.00)	<0.001^a^	-
Gestational age at first antenatal contact, median (IQR)	14 weeks (IQR: 10-17)	16 weeks (IQR: 12-18)	-	-	14 weeks (IQR: 10-18)	<0.001^a^	-
Maternal age, median (IQR)	27 years (IQR: 22-35)	30 years (IQR: 26-35)	-	-	28 years (IQR: 23-35)	<0.001^a^	-
**Categorical variables**							
MAP ≥89 mmHg	106 (27.3%)	132 (68.0%)	-	-	238 (40.9%)	<0.001^b^	-
Preeclampsia in previous pregnancy (ies) ^d^	29 (14.1%)	70 (49.6%)	28 (15.9%)	71 (41.8%)	99 (28.6%)	<0.001^b^	< 0.001^b^
Chronic hypertension	23 (5.9%)	75 (38.7%)	9 (2.6%)	89 (37.4%)	98 (16.8%)	<0.001^b^	< 0.001^b^
Nulliparity	211 (54.4%)	69 (35.6%)	192 (55.8%)	88 (37.0%)	280 (48.1%)	<0.001^b^	< 0.001^b^
Aspirin prophylaxis	26 (6.7%)	28 (14.4%)	20 (5.8%)	34 (14.3%)	54 (9.3%)	0.002^b^	< 0.001^b^
History of preeclampsia in sister/mother	31 (8.0%)	27 (13.9%)	27 (7.8%)	31 (13.0%)	58 (10.0%)	0.024^b^	0.040^b^
Kidney disease	0 (0.0%)	4 (2.1%)	0 (0.0%)	4 (1.7%)	4 (0.7%)	0.012^c^	0.028^c^
Age ≥35 years	105 (27.1%)	60 (30.9%)	85 (24.7%)	80 (33.6%)	165 (28.4%)	0.329^b^	0.019^b^
Pregestational diabetes mellitus	9 (2.3%)	3 (1.5%)	3 (0.9%)	9 (3.8%)	12 (2.1%)	0.759^c^	0.019^c^
Calcium prophylaxis	96 (24.7%)	62 (32.0%)	91 (26.5%)	67 (28.2%)	158 (27.1%)	0.065^b^	0.651^b^
Interpregnancy interval >10 years ^d^	28 (13.7%)	18 (12.8%)	24 (13.6%)	22 (12.9%)	46 (13.3%)	0.81^b^	0.849^b^
Low income	328 (84.5%)	157 (80.9%)	295 (85.8%)	190 (79.8%)	485 (83.3%)	0.271^b^	0.059^b^
Previous adverse outcome/SGA infant ^d^	121 (59.3%)	75 (53.2%)	104 (59.1%)	92 (54.4%)	196 (56.8%)	0.259^a^	0.383^b^
Assisted reproductive technology	3 (0.8%)	2 (1.0%)	2 (0.6%)	3 (1.3%)	5 (0.9%)	1.000^c^	0.404^c^
Multifetal gestation	30 (7.7%)	9 (4.6%)	25 (7.3%)	14 (5.9%)	39 (6.7%)	0.160^b^	0.511^b^
Autoimmune disease	2 (0.5%)	1 (0.5%)	2 (0.6%)	1 (0.4%)	3 (0.5%)	1.000 ^c^	1.000^c^

a: Mann-Whitney U test; b: Pearson Chi square test; c: Fisher's exact test; d: N= 345 Continuous variables presented as median (IQR); categorical variables as n (%) EOPE: early-onset preeclampsia; MAP: mean arterial pressure; SGA: small for gestational age

**Predictive performance of mean arterial pressure for early-onset preeclampsia:** the AUROC for average MAP was 0.783 (95% CI: 0.743-0.824; p <0.001). Using the predefined threshold of MAP ≥89 mmHg, performance metrics were as follows: sensitivity 68.04%, specificity 72.68%, positive predictive value (PPV) 55.46%, negative predictive value (NPV) 81.98%, and overall accuracy 71.13%.

**Added predictive value of maternal risk factors:** the AUROC increased from 0.783 (MAP alone) to 0.810 (95% CI: 0.773-0.848; p <0.001) in the combined model. Figure 2 shows the discriminative ability of MAP alone and MAP ≥89 mmHg combined with maternal risk factors in predicting EOPE.

**Figure 2:**
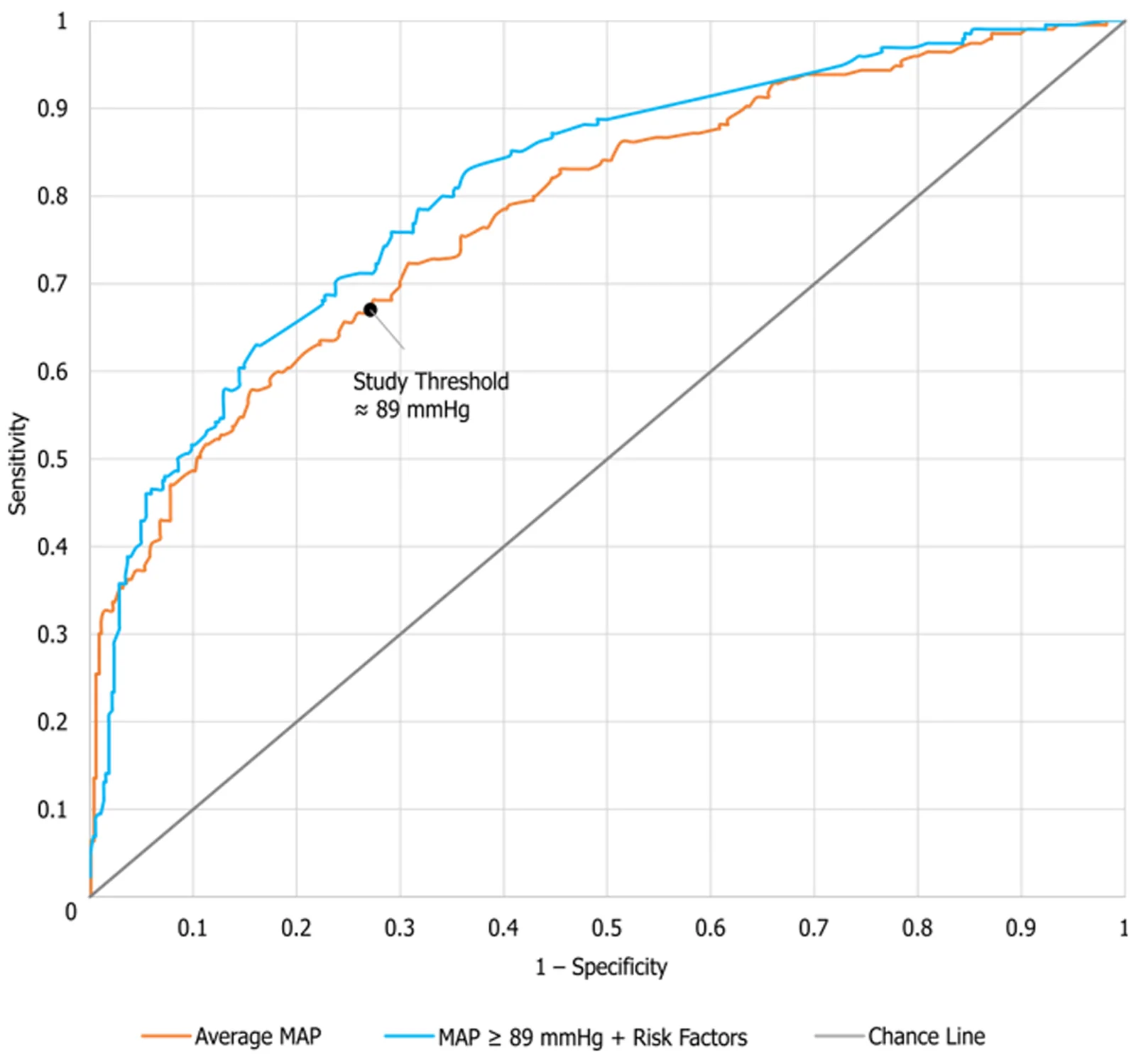
receiver operating characteristic curves for mean arterial pressure alone and combined with maternal risk factors before 20+0 weeks in predicting early-onset preeclampsia. The 89-mmHg threshold is indicated on the mean arterial pressure curve

**Predictive performance of mean arterial pressure across maternal risk subgroups:** the performance of MAP ≥89 mmHg was consistent across maternal risk subgroups. Sensitivity ranged from 52.94% to 82.86% (mean: 69.09% ± 7.03%; median: 67.80%, IQR: 66.67%-74.40%), while specificity ranged from 55.17% to 76.78% (median: 72.61%, IQR: 69.52%-73.78%). Positive predictive value ranged from 42.28% to 81.69% (median: 55.30%, IQR: 54.41%-60.00%), while NPV ranged from 57.14% to 85.88% (median: 81.87%, IQR: 80.62%-83.28%). Overall accuracy ranged from 65.22% to 74.75% (median: 71.20%, IQR: 70.33%-72.14%). Crude odds ratios for EOPE across maternal risk subgroups ranged from 3.60 to 9.13 (mean: 5.75 ± 1.29; median: 5.74, IQR: 5.22-6.12). Table 2, Table 3, and Table 4 summarize the subgroup predictive performance.

**Table 2:** predictive performance of mean arterial pressure ≥89 mmHg before 20+0 weeks for early-onset preeclampsia, stratified by maternal medical conditions and medications

Variable	MAP (mmHg)	No EOPE	EOPE	Sensitivity	Specificity	PPV	NPV	Accuracy	OR (95% CI)	P-value
Overall	<89	282	62	68.04%	72.68%	55.46%	81.98%	71.13%	5.66 (3.89-8.24)	<0.001^a^
	≥89	106	132							
**Calcium prophylaxis**										
No	<89	214	39	70.45%	73.29%	54.39%	84.58%	72.41%	6.54(4.15-10.31)	<0.001^a^
	≥89	78	93							
Yes	<89	68	23	62.90%	70.83%	58.21%	74.73%	67.72%	4.12 (2.09-8.11)	<0.001^a^
	≥89	28	39							
**Aspirin prophylaxis**										
No	<89	269	55	66.87%	74.31%	54.41%	83.02%	71.97%	5.84 (3.91-8.71)	<0.001^a^
	≥89	93	111							
Yes	<89	13	7	75.00%	50.00%	61.76%	65.00%	62.96%	3.00 (0.95-9.48)	0.057^a^
	≥89	13	21							
**Pregestational diabetes mellitus**										
No	<89	279	62	67.54%	73.61%	56.33%	81.82%	71.58%	5.81 (3.97-8.48)	<0.001^a^
	≥89	100	129							
Yes	<89	3	0	100.00%	33.33%	33.33%	100.00%	50.00%	-	0.509^b^
	≥89	6	3							
**Kidney disease**										
No	<89	282	62	67.37%	72.68%	54.70%	81.98%	70.93%	5.49 (3.77-8.00)	<0.001^a^
	≥89	106	128							
Yes	<89	0	4	-	-	-	-	-	-	-
	≥89	0	0							
**Chronic hypertension**										
No	<89	279	56	52.94%	76.44%	42.28%	83.28%	70.66%	3.65 (2.37-5.63)	<0.001^a^
	≥89	86	63							
Yes	<89	3	6	92.00%	13.04%	77.53%	33.33%	73.47%	1.73 (0.40-7.52)	0.434^a^
	≥89	20	69							
**Autoimmune disease**										
No	<89	280	62	67.88%	72.54%	55.27%	81.87%	70.98%	5.58 (3.83-8.13)	<0.001^a^
	≥89	106	131							
Yes	<89	2	0	100.00%	100.00%	100.00%	100.00%	100.00%	-	0.333^b^
	≥89	0	1							

a: Pearson Chi-square test; b: Fisher's exact test; PPV:positive predictive value; NPV: negative predictive value; MAP: mean arterial pressure; EOPE: early-onset preeclampsia

**Table 3:** predictive performance of mean arterial pressure ≥89 mmHg before 20+0 weeks for early-onset preeclampsia, stratified by maternal sociodemographic characteristics

Variable	MAP (mmHg)	No EOPE	EOPE	Sensitivity	Specificity	PPV	NPV	Accuracy	OR (95% CI)	P-value
Overall	<89	282	62	68.04%	72.68%	55.46%	81.98%	71.13%	5.66 (3.89-8.24)	<0.001^a^
	≥89	106	132							
**Age ≥35 years**										
No	<89	209	50	62.69%	73.85%	53.16%	80.62%	70.26%	4.75 (3.06-7.36)	<0.001^a^
	≥89	74	84							
Yes	<89	73	12	80.00%	69.52%	60.00%	85.88%	73.33%	9.13 (4.28-19.45)	<0.001^a^
	≥89	32	48							
**Low income**										
No	<89	40	9	75.68%	66.67%	58.33%	81.63%	70.10%	6.22 (2.47-15.66)	<0.001^a^
	≥89	20	28							
Yes	<89	242	53	66.24%	73.78%	54.74%	82.03%	71.34%	5.52 (3.66-8.34)	<0.001 ^a^
	≥89	86	104							
**History of preeclampsia (sister/mother)**										
No	<89	264	53	68.26%	73.95%	55.07%	83.28%	72.14%	6.11 (4.09-9.13)	<0.001^a^
	≥89	93	114							
Yes	<89	18	9	66.67%	58.06%	58.06%	66.67%	62.07%	2.77 (0.95-8.09)	0.060^a^
	≥89	13	18							

a: Pearson Chi-square test; PPV: positive predictive value; NPV: negative predictive value; MAP: mean arterial pressure; EOPE: early-onset preeclampsia

**Table 4:** predictive performance of mean arterial pressure ≥89 mmHg before 20+0 weeks for early-onset preeclampsia, stratified by maternal clinical characteristics

Variable	MAP (mmHg)	No EOPE	EOPE	Sensitivity	Specificity	PPV	NPV	Accuracy	OR (95% CI)	P-value
**Overall**	<89	282	62	68.04%	72.68%	55.46%	81.98%	71.13%	5.66 (3.89-8.24)	<0.001^a^
	≥89	106	132							
**Nulliparity**										
No	<89	120	32	74.40%	67.80%	62.00%	78.95%	70.60%	6.12 (3.67-10.20)	<0.001 ^a^
	≥89	57	93							
Yes	<89	162	30	56.52%	76.78%	44.32%	84.38%	71.79%	4.30 (2.42-7.63)	<0.001^a^
	≥89	49	39							
**Interpregnancy interval >10 years**										
No	<89	123	29	76.42%	69.49%	63.51%	80.92%	72.33%	7.38 (4.37-12.48)	<0.001^a^
	≥89	54	94							
Yes	<89	18	6	66.67%	64.29%	54.55%	75.00%	65.22%	3.60 (1.03-12.54)	0.04^a^
	≥89	10	12							
**Previous adverse outcome or SGA infant**										
No	<89	55	17	74.24%	66.27%	63.64%	76.39%	69.80%	5.66 (2.77-11.56)	<0.001^a^
	≥89	28	49							
Yes	<89	86	18	76.00%	71.07%	61.96%	82.69%	72.95%	7.78 (4.02-15.05)	<0.001^a^
	≥89	35	57							
**Assisted reproductive technology**										
No	<89	280	62	67.71%	72.73%	55.32%	81.87%	71.05%	5.59 (3.84-8.15)	<0.001^a^
	≥89	105	130							
Yes	<89	2	0	100.00%	66.67%	66.67%	100.00%	80.00%	-	0.400^b^
	≥89	1	2							
**Preeclampsia in previous pregnancy(ies)**										
No	<89	125	23	67.61%	71.02%	48.48%	84.46%	70.04%	5.12 (2.82-9.27)	<0.001^a^
	≥89	51	48							
Yes	<89	16	12	82.86%	55.17%	81.69%	57.14%	74.75%	5.95 (2.28-15.54)	<0.001^a^
	≥89	13	58							
**Multifetal gestation**										
No	<89	261	58	68.65%	72.91%	56.70%	81.82%	71.45% ;	5.89 (4.00-8.69)	<0.001^a^
	≥89	97	127							
Yes	<89	21	4	55.56%	70.00%	35.71%	84.00%	66.67%	2.92 (0.63-13.46)	0.238^b^
	≥89	9	5							

a: Pearson Chi-square Test; b: Fisher's exact test; PPV: positive predictive value; NPV: negative predictive value; MAP: mean arterial pressure; EOPE: early-onset preeclampsia; SGA: small for gestational age

**Bivariate analysis:** mean arterial pressure ≥89 mmHg was associated with EOPE (OR = 5.66, 95% CI: 3.89-8.24, p <0.001). Prior preeclampsia and chronic hypertension were also strongly associated with EOPE. Aspirin prophylaxis and family history of preeclampsia were associated with EOPE, while nulliparity was associated with lower odds. Table 5 shows the unadjusted associations between maternal risk factors and early-onset preeclampsia.

**Table 5:** unadjusted associations of mean arterial pressure ≥89 mmHg and maternal risk factors before 20+0 weeks with early-onset preeclampsia

Predictor variable	No EOPE (%) (n = 388)	EOPE (%) (n = 194)	P-value^a^	OR	95% CI for OR	
					Lower	Upper
MAP ≥89 mmHg	106 (27.3%)	132 (68.0%)	<0.001	5.66	3.89	8.24
Preeclampsia in previous pregnancy(ies)	29 (14.1%)	70 (49.6%)	<0.001	5.95	3.56	9.94
Chronic hypertension	23 (5.9%)	75 (38.7%)	<0.001	10.00	6.00	16.67
Nulliparity	211 (54.4%)	69 (35.6%)	<0.001	0.46	0.33	0.66
Aspirin prophylaxis	26 (6.7%)	28 (14.4%)	0.002	2.35	1.34	4.13
History of preeclampsia in sister/mother	31 (8.0%)	27 (13.9%)	0.024	1.86	1.08	3.22
Kidney disease^c^	0 (0.0%)	4 (2.1%)	0.012 b	-	-	-
Age ≥35 years	105 (27.1%)	60 (30.9%)	0.329	1.21	0.83	1.76
Pregestational diabetes mellitus	9 (2.3%)	3 (1.5%)	0.759 b	0.66	0.18	2.47
Calcium prophylaxis	96 (24.7%)	62 (32.0%)	0.065	1.43	0.98	2.09
Interpregnancy interval >10 years	28 (13.7%)	18 (12.8%)	0.81	0.92	0.49	1.74
Low income	328 (84.5%)	157 (80.9%)	0.271	0.78	0.49	1.22
Previous adverse outcome/SGA infant	121 (59.3%)	75 (53.2%)	0.259	0.78	0.51	1.20
Assisted reproductive technology	3 (0.8%)	2 (1.0%)	1.000^a^	1.34	0.22	8.07
Multifetal gestation	30 (7.7%)	9 (4.6%)	0.160	0.58	0.27	1.25
Autoimmune disease	2 (0.5%)	1 (0.5%)	1.000^b^	1.00	0.90	11.10

a: Pearson Chi-square test; b: Fisher's exact test; OR:odds ratio; CI: confidence interval; c: Kidney disease produced unstable estimates due to sparse data and was not interpretable; PPV: positive predictive value; NPV: negative predictive value; MAP: mean arterial pressure; EOPE: early-onset preeclampsia; SGA: small for gestational age

**Multivariable analysis:** after adjustment for maternal risk factors, MAP ≥89 mmHg remained an independent predictor of EOPE (aOR = 3.04; 95% CI: 1.96-4.71; p <0.001). Previous preeclampsia and chronic hypertension remained strong independent predictors. Maternal age ≥35 years and pregestational diabetes mellitus (PGDM) were inversely associated with EOPE. Table 6 shows the adjusted associations between maternal risk factors and EOPE.

**Table 6:** adjusted associations of mean arterial pressure ≥89 mmHg and maternal risk factors before 20+0 weeks with early-onset preeclampsia

Predictor variable	aOR	95% CI for aOR		P-value
		Lower	Upper	
MAP ≥89 mmHg	3.04	1.96	4.71	<0.001
Preeclampsia in previous pregnancy(ies)	4.16	2.25	7.68	<0.001
Chronic hypertension	5.17	2.69	9.94	<0.001
Age ≥35 years	0.54	0.31	0.94	0.029
Diabetes mellitus (Pregestational)	0.21	0.05	0.99	0.048
Calcium prophylaxis	1.39	0.85	2.27	0.196
Aspirin prophylaxis	0.81	0.35	1.87	0.627
Nulliparity	0.76	0.44	1.32	0.333
Interpregnancy interval >10 years	1.37	0.60	3.11	0.452
Low income	0.85	0.49	1.48	0.568
History of preeclampsia in sister/mother	1.46	0.75	2.85	0.271
Previous adverse pregnancy outcome/SGA infant	0.72	0.44	1.18	0.186
Assisted reproductive technology	1.32	0.13	13.67	0.819
Multifetal gestation	0.71	0.29	1.75	0.462
Autoimmune disease	1.29	0.09	17.98	0.852
Kidney disease^a^	265580457.3	-		0.999

Note: all primigravida NA recoded to No; aOR: adjusted odds ratio; CI: confidence interval; a: Kidney disease produced unstable estimates due to sparse data and was not interpretable; SGA: small for gestational age; MAP: mean arterial pressure

## Discussion

**Study population and overview:** this study evaluated the predictive performance of mean arterial pressure (MAP) ≥89 mmHg measured before 20+0 weeks of gestation for early-onset preeclampsia (EOPE) in 582 women with at least one risk factor for preeclampsia (194 cases and 388 controls). Early-onset preeclampsia cases had a higher burden of established risk factors, including previous preeclampsia, chronic hypertension, aspirin use, kidney disease, and family history of preeclampsia (sister/mother), consistent with known epidemiological patterns of preeclampsia risk [1-3,13-18]. Similarly, MAP ≥89 mmHg clustered with classical risk factors, including previous preeclampsia, chronic hypertension, aspirin prophylaxis, family history of preeclampsia (sister/mother), kidney disease, maternal age ≥35 years, and pregestational diabetes mellitus (PGDM), reflecting underlying vascular susceptibility and aggregation of cardiometabolic risk factors [13].

**Predictive performance of mean arterial pressure for early-onset preeclampsia:** mean arterial pressure was consistently higher among EOPE cases than controls, supporting previous evidence that early pregnancy MAP is a strong indicator of subsequent preeclampsia risk [4,18-20], although it has limited ability to discriminate between women who will and will not develop the disease [5]. Mean arterial pressure demonstrated fair discriminative ability for EOPE, with an AUROC of 0.783 [12]. This aligns with previous research where early pregnancy blood pressure-based prediction models yielded AUROC values ranging from 0.619 to 0.860 [5,11,18-23]. The predefined threshold of 89 mmHg used in this study aligns with previously documented thresholds between 88 and 93.67 mmHg [4,5,18,20,21,24,25]. At this cut-off, moderate predictive performance was achieved, with fair sensitivity (68.04%), good specificity (72.68%), and a high negative predictive value (NPV) (81.98%). The high negative predictive value may be particularly useful in clinical settings, as EOPE can be reliably ruled out in women with normal MAP values. Similar predictive performance for early-pregnancy MAP has been reported in comparable studies [5,19,20,25].

**Added predictive value of maternal risk factors:** the addition of maternal risk factors increased the AUROC from 0.783 to 0.810. This indicates that while meaningful predictive data is provided by MAP alone, its performance is enhanced when combined with maternal risk factors. This observation aligns with prior research demonstrating improved prediction when early-pregnancy MAP is integrated with maternal characteristics [11,17,22,23,26].

**Predictive performance of mean arterial pressure across maternal risk subgroups:** the predictive performance of MAP ≥89 mmHg remained stable across maternal risk subgroups, with fair sensitivity (median: 67.80%, IQR: 66.67%-74.40%), good specificity (median: 72.61%, IQR: 69.52%-73.78%), and a high NPV (median: 81.87%, IQR: 80.62%-83.28%). Although minor variations in predictive metrics were observed, overall performance was consistent, supporting the robustness of MAP as a predictor of EOPE. This is consistent with previous research demonstrating similar predictive performance of MAP across maternal risk groups [21]. The NPV remained consistently high across subgroups, supporting its clinical potential for ruling out EOPE. Detailed performance metrics for each subgroup are provided in Table 2, Table 3, and Table 4.

**Mean arterial pressure ≥89 mmHg as an independent predictor of early-onset preeclampsia:** mean arterial pressure ≥89 mmHg remained independently associated with EOPE (aOR = 3.04), consistent with previous studies concluding that MAP is a useful predictive parameter for preeclampsia [4,11,18,21,24]. Prior preeclampsia and chronic hypertension were also independently predictive of EOPE, aligning with studies that have classified these as high-risk factors for preeclampsia. Maternal age ≥35 years and pregestational diabetes mellitus (PGDM) were inversely associated with EOPE in the adjusted model, contrary to established evidence [1-3,13-18]. These findings may be explained by residual confounding, selection bias related to the inclusion of at-risk women, or small subgroup sizes, especially among women with PGDM. Greater healthcare engagement among older women may also have contributed to earlier detection and management, thereby influencing the observed associations.

**Study strengths:** this case-control study involved 582 participants in a 1: 2 ratio, which provided the study with sufficient statistical power to provide significant associations. Also, this is one of the few studies that have been conducted to investigate the predictive ability of MAP in predicting EOPE in a Kenyan population, which is a pressing need in the country and LMICs at large. Moreover, the stratified analyses and multivariate logistic regression (which included all variables independent of bivariate significance) were useful in minimizing bias, confounding, and effect modification, which enhanced internal and external validity by accounting for potential confounding. Additionally, the use of a clearly defined MAP threshold (≥89 mmHg), and an early gestational window (before 20+0 weeks) provides practical clinical guidance, as this can be obtained from routine antenatal data.

**Study limitations:** the results of this study may not be as broadly applicable in other contexts due to the fact that it was a single-center study carried out at Kenyatta National Hospital (KNH). Unstable estimates were also produced by sparse data within uncommon subgroups, such as women with kidney disease, among others. Second, only women with known preeclampsia risk factors were included in the study, the results might not apply directly to populations without risk factors for preeclampsia. Third, there was an inherent risk of selection bias in the case-control design, and both positive and negative predictive values may have been impacted since they relied on the prevalence of EOPE in the population. Lastly, blood pressure values were extracted from ANC booklets, and different blood pressure measurement techniques across clinics and visits prior to 20 weeks may have introduced some variability. Additionally, the retrospective nature of the data made it challenging to sufficiently demonstrate temporal connections between the onset of EOPE and MAP ≥89 mmHg. To minimize bias, we used a clearly defined MAP threshold, conducted stratified analyses, and included all study variables in the multivariable logistic regression.

**Clinical implications of mean arterial pressure screening before 20+0 weeks of gestation:** for women at risk of EOPE, MAP is a simple, feasible, and reproducible screening tool. This study´s MAP threshold demonstrated moderate sensitivity and specificity, high NPV, and a positive association with EOPE, and therefore, is useful as a rule-out test, which could reduce unnecessary intensive monitoring for low-risk women. Additionally, early risk stratification allows the timely implementation of preventive measures such as aspirin or calcium prophylaxis, potentially reducing the risk of EOPE and its complications. Finally, incorporating MAP screening into national antenatal care protocols is a scalable and economical policy-level intervention that has the potential to enhance maternal and fetal outcomes, particularly in settings with limited resources.

## Conclusion

The findings of our study show that MAP ≥89 mmHg measured prior to 20+0 weeks of gestation can be useful as an independent and early predictor of EOPE in at-risk women. Since it is simple, affordable, and easy to measure, MAP is a practical tool that can be incorporated into routine antenatal care, especially in low-resource settings where access to advanced laboratory or imaging tests may be limited. Mean arterial pressure-based screening, including maternal characteristics, could help identify women at risk and inform early decision-making in managing these patients.

***What is known about this topic***

- *Early-onset preeclampsia (EOPE) is associated with significant maternal and perinatal morbidity and mortality, particularly in low- and middle-income countries (LMICs);*
- *Mean arterial pressure (MAP) measured in the first or second trimester has been proposed as a potential predictor of preeclampsia;*
- *Evidence on the predictive performance of early MAP measurements (before 20+0 weeks of gestation) remains limited, particularly in African LMIC populations*.

***What this study adds***

- *Demonstrates that MAP ≥89 mmHg before 20+0 weeks of gestation is independently associated with EOPE among at-risk Kenyan women, and shows that MAP has fair discriminative ability for EOPE (AUROC =0.783), which improves when combined with maternal risk factors (AUROC = 0.810);*
- *Suggests a consistently high negative predictive value (NPV) across maternal risk subgroups, supporting MAP as a useful rule-out tool;*
- *Provides context-specific evidence supporting the integration of early MAP measurement into routine antenatal care in LMIC settings*.
